# NADPH Oxidase Isoform 2 (NOX2) Is Involved in Drug Addiction Vulnerability in Progeny Developmentally Exposed to Ethanol

**DOI:** 10.3389/fnins.2017.00338

**Published:** 2017-06-14

**Authors:** Marcela L. Contreras, Erwin de la Fuente-Ortega, Sofía Vargas-Roberts, Daniela C. Muñoz, Carolina A. Goic, Paola A. Haeger

**Affiliations:** Departamento de Ciencias Biomédicas, Facultad de Medicina, Universidad Católica del NorteCoquimbo, Chile

**Keywords:** drug addiction, fetal programming, ROS, superoxide, alcohol, development, oxidative stress, NMDAR

## Abstract

Ethanol exposure increases oxidative stress in developing organs, including the brain. Antioxidant treatment during maternal ethanol ingestion improves behavioral deficits in rodent models of fetal alcohol spectrum disorder (FASD). However, the impact of general antioxidant treatment in their adult offspring and the Specific Reactive Species (ROS)-dependent mechanism, are not fully understood. We hypothesized that pre and early postnatal ethanol exposure (PEE) modifies redox homeostasis, in particular NOX2 function during reward signaling in the mesocorticolimbic pathway, which reinforces the effects of alcohol. We developed a FASD rat model which was evaluated during adolescence (P21) and adulthood (P70). We first studied whether redox homeostasis is affected in PEE animals, by analyzing mRNA expression of SOD1, CAT, and Gpx1. We found that PEE reduced the mRNA levels of these three anti-oxidant enzymes in PFC and HIPP at P21 and in the VTA at P70. We also analyzed basal mRNA and protein expression of NOX2 subunits such as gp91phox, p22 phox, and p47 phox, in mesocorticolimbic brain areas of PEE rat brains. At P21, gp91 phox, and p47 phox levels in the VTA were decreased. At P70, gp91 phox mRNA levels was decreased in HIPP and both mRNA and protein levels were decreased in PFC. Since NOX2 is regulated by the N-methyl-D-aspartate Receptor (NMDAR), we analyzed NMDAR mRNA expression and found differential expression of NMDAR subunits (NR1 and NR2B) in the PFC that was age dependent, with levels decreased at P21 and increased at P70. The analysis also revealed decreased NR2B mRNA expression in HIPP and VTA at P70. Offspring from maternal ethanol users consumed 25% more ethanol in a free choice alcohol consumption test than control rats, and showed place preference for an alcohol-paired compartment. *In vivo* inhibition of NOX2 using apocynin in drinking water, or infusion of blocked peptide gp91 phox ds in the VTA normalized alcohol place preference, suggesting that NOX2 plays an important role in addictive like behavior. Taken together, PEE significantly affects the expression of antioxidant enzymes, NOX2, NMDAR in an age, and brain region dependent manner. Moreover, we demonstrate that NOX2 regulates alcohol seeking behavior.

## Introduction

Alcohol and tobacco are legal substances used by thousands of people worldwide daily. Abuse of these substances can lead to serious long lasting effects to human health, as well as high social and economic impacts. During pregnancy, alcohol ingestion may cause severe birth defects or intellectual impairment in offspring (Alfonso-Loeches and Guerri, 2011). Dysfunctions associated with prenatal exposition to ethanol are collectively termed FASD and are characterized by a range of developmental, cognitive, and neurobehavioral abnormalities (Jones and Smith, 1973; Clarren and Smith, 1978). Moderate maternal drinking (1–2 drinks per day) does not typically cause full-blown FAS; however, it is associated with cognitive and behavioral alterations in the offspring, probably expressed during demanding situations (Streissguth et al., 1990, 1994; Willford et al., 2004).

According to the last systematic review and meta-analysis publication, (Popova et al., 2017), in the worldwide population FAS effects approximately 14.6 per 10,000 people, increasing in regions with binge drinking behavior such as the European region (37.4 per 10,000 people). Moreover, an extensive study revealed that 1% of pregnant women in Santiago of Chile consumed at least 4 drinks per day (101 from 9,628). This study revealed that 44% (22/50) of alcohol-exposed children, compared to 13.6% (6/44) of unexposed, present one or more functional central nervous system abnormality (Kuehn et al., 2012). However, since there is no existing information about offspring born under these conditions, the prevalence of neurobiological abnormalities of children prenatally exposed to alcohol is probably higher than that of individuals diagnosed with FASD.

Early alcohol exposition increases the probability of drug abuse during adulthood, similar to the behavioral alterations described in offspring that were prenatally exposed to illicit drugs (Baer et al., 2003; Malanga and Kosofsky, 2003). Also, clinical studies report that chronic fetal ethanol exposure increases the risk of ethanol and other drug dependence later in life (Famy et al., 1998; Yates et al., 1998). Preclinical studies in infant (Miranda-Morales et al., 2014) or adolescent rats (Pautassi et al., 2012; Fabio et al., 2013) showed that exposure to ethanol during late gestation promoted ethanol-mediated reinforcement. Additionally, 2-month-old rats that were exposed to ethanol during gestation and nursing also showed increased ethanol consumption, ethanol conditioning place preference, and enhanced locomotor activity triggered by psychostimulants and ethanol (Barbier et al., 2009). Together, these studies confirmed that PEE causes addictive-like behaviors during adulthood.

The effect of alcohol at the molecular level has been extensively studied and shown to produce ROS imbalance. Several studies suggested that ethanol consumption produces oxidative stress in pregnant mothers and offspring brains (Brocardo et al., 2012), as cellular redox state is abnormally expressed at 90 days old (decreased GSH and increased SOD) (Dembele, 2006). Furthermore, abnormal redox state correlates with memory deficits (Vink et al., 2005) and antioxidant treatment during maternal ethanol ingestion improves behavioral deficits in rodent models of FASD (Brocardo et al., 2012). However, little is known about the role of ROS in addictive processes triggered by *in utero* alcohol exposure. Nevertheless, recent evidence shows that oxidation of redox sensors (S-Glutathionlylation and S-Nitrosylation) correlates with models of substance abuse (Uys et al., 2014; Jang et al., 2016). Treatments with N-acetyl cysteine (NAC), a potent antioxidant, decrease cocaine relapse in human as well in cocaine dependent-animal models (Mardikian et al., 2007). Also, NAC is a cystine prodrug that promotes cystine/glutamate exchange, thereby raising extracellular glutamate levels in the nucleus accumbens of cocaine seeking animals (Okamoto et al., 2006). In humans, NAC decreases glutamate levels in the dorsal anterior cingulate cortex of cocaine dependent patients (Schmaal et al., 2012); possibly a mechanism contributing to its clinical efficacy (Mardikian et al., 2007). Thus, these data suggest that the addictive process induced by *in utero* alcohol exposure could involve an imbalance of ROS.

The influence of ROS on the regulation of synaptic plasticity and memory has been extensively studied (Massaad and Klann, 2011). One important source of ROS is the membrane complex NOX2 (NADPH oxidases, postsynaptic superoxide generator), which through NMDAR regulation can modulate synaptic plasticity and memory formation (Kishida et al., 2005, 2006; Kishida and Klann, 2007). In addition, reciprocal regulation of NOX2 by the NMDA receptor was observed in *in vitro* studies (Brennan et al., 2009). The NOX family of proteins is an enzymatic complex that transfers electrons across biological membranes, producing superoxide; the most active ROS (Bedard and Krause, 2007). Neurons predominantly express NOX2 (gp91phox) (Tammariello et al., 2000) at the synapse (Tejada-Simon et al., 2005; Todkar et al., 2015). NOX2 enzymes are composed of catalytic (gp91phox) and regulatory subunits, and their activation depends on the formation of an active complex, composed of membrane factor p22 phox and cytosolic factors p40 phox, p47 phox, and p67 phox, which are recruited to the membrane by calcium and PKC signaling (Bedard and Krause, 2007). Recent evidence showed that 7 days old mice exposed to ethanol increased NOX2 activity in the cerebral cortex similar to effect in cultured neuronal cells (Wang et al., 2012). Moreover, embryos from an acute animal model of PEE presented increased NOX activity in the brain (Dong et al., 2010). We hypothesized that PEE modifies NOX2 function, impacting reward signaling in the mesocorticolimbic pathway, which may contribute to reinforcing the effects of alcohol. We tested the role of NOX on alcohol seeking behavior of animals exposed to ethanol *in utero*. We used behavioral animal assays, RT-qPCR, and immunoblot detection to evaluate the effects of ethanol on the expression of redox enzyme systems (CAT, SOD, Gpx1, and NOX2) and glutamatergic NMDAR subunits (NR1, NR2B) in the rat mesocorticolimbic brain areas in a model of PEE. We found that several enzymes involved in redox homeostasis -CAT, SOD, Gpx1, and NOX2- and NMDAR subunits were deregulated in PEE rats. Moreover, inhibition of NOX2, specifically in VTA, could rescues ethanol seeking behavior in adult rats in PEE conditions (P70). These findings suggest a new therapeutic target for preventing addictive behavior in vulnerable individuals and treatment of patients with problematic alcohol consumption or other drug abuse.

## Materials and methods

### Animals and treatment

Pregnant Sprague-Dawley rats were exposed to water or ethanol (10% v/v) both sweetened with 64 mg/l of sucralose (Daily). The consumption of liquid and food was monitored during all treatment. Ethanol solution consumption was started on day 5 ± 2 days of gestation until 1 week after the offspring were born (P7). A daily liquid complement (tap water) was administered for 2 h to pregnant mothers consuming ethanol. After the ethanol protocol, rats were left with food, and water *ad libitum*. Offspring were weaned at 21 days after birth (P21) and separated by sex.

Offspring were weighed at P1 and P7. In order to calculate the average maternal weight during treatment, the weight was considered in the initial and final state of the treatment.

Blood was extracted from the mother's tail 12 h after light turn off and serum was used to quantify alcohol concentration by the QuantiChromTM Ethanol Assay Kit (DIET-500) (BioAssay Systems).

In all the experiments, one sample from an offspring of each litter was used. The sample may be an average of 2 or 3 offspring per litter. Each experiment was repeated at least three times with different litters.

Protocols for rat handling were carried out in accordance with the recommendations of the Assessor Committee in Bioethics guidelines from the National Fund for Scientific and Technological Development (FONDECYT, Chile) and approved by the Bioethic, Scientific, and Animal Care and Use Committee of the Universidad Católica del Norte, Chile.

### Brain sample extraction

Different brain samples were extracted by microdissection. The brain region enriched of PFC (contained prelimbic and infralimbic PFC) and VTA was extracted from limited slices approximately from 4.2 to 2.7 mm and −5.2 to −6.8 mm from bregma, respectively, according to the Paxinos and Watson (1998). From the same brain, whole HIPP was also extracted. Samples were extracted from P21 and P70 male offspring, which were exposed or not to ethanol *in utero*. Samples were collected on dry ice and stored at −80°C until processed.

### Western blot

We followed the protocols described previously (Adasme et al., 2011). Tissues were homogenized with RIPA buffer (1% NP40, 0.5% Deoxycholate, 0.1% SDS, 150 mM NaCl, 50 mM Tris-Cl, pH8.0), 10–30 μg of protein was suspended in 3X loading buffer and denatured at 80°C for 10 min. Proteins were separated by sodium dodecyl sulfate polyacrylamide gel electrophoresis (SDS-PAGE), and transferred to 0.2 μm nitrocellulose membranes (Protran, Whatman, Germany) for 90 min at 450 mA. The membranes were blocked with blocking buffer containing 3% non-fat milk in TBS1X buffer (20 mM Tris-HCl and 137 mM NaCl) for 1 h at room temperature and incubated for 18 h at 4°C with the following primary antibodies; 1:5000 anti-gp91 (Abcam), 1:10.000 anti-β-actin (Sigma-Aldrich). The membranes were washed three times with 0.1% tween-TBS1X at room temperature and incubated with secondary antibodies conjugated to horseradish peroxidase (HRP) (anti-rabbit HRP or anti-mouse HRP, both from Cell Signaling) dissolved in 3% non-fat milk-TBS1X for 1 h at room temperature. The blots were washed three times with 0.1% Tween-TBS1X exposed to chemiluminescent substrate (198 μM of p-coumaric acid and 1.25 mM of 3-aminophtalhydrazina, in 10 mM Tris-Cl pH8.5) for 1 min and scanned with a C-digit blot scanner (LI-COR). The bands were quantified by ImageJ software (National Institutes of Health, USA) and normalized to β-actin.

### RNA isolation and RT-PCR

Total RNA was extracted from tissues by the phenolic extraction method with TRIzol (TRIzol® Reagent, Invitrogen™ Life Technologies, USA). 50–100 mg of tissue sample from PFC, VTA and HIPP were homogenized with TRIzol. RNA samples were treated with 1 Unit of DNase (Turbo DNA-free™ Kit, Life-Technologies) and absorbance ratios close to 2 (260/280 nm) measured by a spectrophotometer (Ultrospec-1000, Pharmacia Biotech) were considered optimal. cDNA was synthesized from total RNA by reverse transcription assay as previously described (Adasme et al., 2011). RNA was denatured at 70°C for 5 min and mixed with a final concentration of 0.02 μg/μl oligo-dT, 2.4 mM MgCl_2_, 0.4 mM deoxynucleotide triphosphate (dNTPs), 1 μl reverse transcriptase (Improm II ™, Promega, USA), buffer 1X (250 mM Tris-HCl, pH 8.3, 375 mM KCl, and 15 mM MgCl_2_) and nuclease-free water, with a final reaction volume of 20 μl that was incubated for 5 min at 25°C, 1 h at 42°C, and 15 min at 70°C. As a control, the same reaction mix for each sample was performed without reverse transcriptase. All tubes were diluted twice with nuclease-free water and 1 μl of the final dilution was amplified by real time PCR. Quantitative real-time PCR was performed using Real-Time PCR Systems (StepOne™, Applied Biosystems). In the reaction we used 50 nM of each primer (Table 1) and Sybr green kit (Kapa Sybr®Fast, Biosystems, USA).

**Table 1:** Primer used for qPCR.

**Gen**	**Forward/Reverse (5′-3′)**
GAPDH	AACGACCCCTTCATTGAC/TCCACGACATACTCAGCAC
β-actin	TCTACAATGAGCTGCGTGTG/TACATGGCTGGGGTGTTGAA
gp91 phox	TGACTCGGTTGGCTGGCATC/CGCAAAGGTACAGGAACATGGG
P22 phox	TCTATTGTTGCAGGAGTGCTCATCT/TTGGTAGGTGGCTGCTTGATG
P47 phox	TCACCGAGATCTACGAGTTC/ATCCCATGAGGCTGTTGAAGT
NR1	CTTCCTCCAGCCACTACCC/AGAAAGCACCCCTGAAGCAC
NR2B	TGAGTGAGGGAAGAGAGAGAGG/ATGGAAACAGGAATGGTGGA
SOD1	CGGTGCAGGGCGTCATTCACTT/CTCTTCATCCGCTGGACCGCC
CAT	ACTGGGACCTCGTGGGAAAC/TCTGGAATCCCTCGGTCGCT
Gpx1	CACCACGACCCGGGACTACA/AGGTAAAGAGCGGGTGAGCC

The PCR conditions were, initial denaturation for 10 min at 95°C followed by 40 cycles of denaturation at 95°C for 15 s, and annealing/extension at 60°C for 30 s. Gene expression levels were normalized to the expression of housekeeping genes, β-actin or GAPDH, according the PCR efficiency. Efficiency was determined using serial dilutions of cDNAs obtained from brain cortex mRNA and calculated based on the slope of the curve. Relative mRNA levels between PEE and control animals were calculated according to the 2^−ΔΔCT^ method, using the equation 2^−ΔΔCT^ = (CT gene of interest- CT housekeeping gene)_PEE_ -(CT gene of interest-CT housekeeping gene)_Control_, (Livak and Schmittgen, 2001; Pfaffl, 2001). 1–3 animals of each litter were analyzed and values were averaged per litters, and a minimum of 3 litters were analyzed.

### Conditioned place preference (CPP)

We followed the protocols previously described by several authors (Barbier et al., 2009; Quintanilla and Tampier, 2011; Whitaker et al., 2013). For conditioning, a CPP box consisting of two distinct compartments was used. One compartment had a mesh floor with horizontal white lines, while the other had a grid floor with verticals white lines. Using blind selection of animals, rats were first subjected to a pre-test, in which they were allowed to freely explore the CPP box for 10 min. The percentage of time spent in each compartment was determined, and any rats that displayed >60% initial preference for either compartment during the pre-test were not used for conditioning. For ethanol CPP, rats were subjected to 2 days of conditioning, where they were given an injection of ethanol (1 gr/kg, i.p. 30% v/v diluted in saline) and confined to one compartment for 10 min, 6 h after the first injection rats received a saline injection and were then confined to the opposite compartment for 10 min. A 10 min post-test was performed 1 day after the last conditioning session. Preference for the ethanol-paired side was defined as the % of time spent in the ethanol-paired compartment in regards to the total time (10 min) on day 1 (before conditioning) and day 4 (after conditioning). The CPP score was determined by calculating the preference ratio for the ethanol-paired side in the pre-test compared to the post-test. Housing conditions were maintained throughout the CPP experiments.

### Free choice protocol (two-bottle choice protocol)

We followed the protocol described previously (Phillips et al., 1998). Two to four rats of P70 were housed and acclimated to our drinking room for at least 2 weeks before the study. During the free choice protocol, isolated animals had access to tap water or a 10% ethanol solution. Fluid consumption was evaluated by reading fluid volumes in the morning (9:30 A.M.) or after 24 h and adjusted for leakage by using volume changes from tubes on empty control cages. The animals were weighed to calculate the grams of water or ethanol consumption (g/kg). The ratio between water vs. ethanol solution consumption was calculated.

### Stereotaxic surgery

Sprague-Dawley rats were anesthetized with a mixture of air and isoflurane and placed in a stereotaxic frame. Cannula (Plastic One) was implanted unilaterally into VTA [coordinates: AP +3.4 (Lambda); L -2.1; DV-7.2, 12 degrees angle] (Rivera-Meza et al., 2014) according to the atlas of Paxinos and Watson (1998) (Paxinos and Watson, 1998). After recovery, animals were transferred to individual cages at the animal station, with access to water and food *ad libitum*. One week after surgery, the rats were infused each day with 100 ng of gp91 ds–tat (Anaspec) 20 min before exposure to CPP assay.

Apocynin (4′-Hydroxy-3′-methoxyacetophenone, Sigma) was added to drinking water at 5 mM and treatment was performed for 5 weeks (P30–P70) (Furukawa et al., 2004; Harraz et al., 2008; Espinosa et al., 2013).

### Statistical analysis

Data from groups (Control and PEE) are presented as means ± SEM. Differences in mean values were compared by unpaired *t*-test. For mRNA expression of NOX2, NMDAR and antioxidant enzymes expression levels a paired student *t*-test was used. *p* < 0.05 was considered statistically significant.

## Results

### PEE increases ethanol-seeking behavior

To evaluate the effects of PEE, we developed a rat model of PEE using forced ethanol consumption. The pregnant mother's daily consumption is shown in Supplementary Figure 1. On average mothers consumed 34.8 ± 2.6 ml/day of 10% ethanol + 64 mg/l sucralose solution during 16.71 ± 1.98 days, vs. 55.4 ± 2.8 ml/day of water + 64 mg/l sucralose in control rats for 17.42 ± 1.98 days during the pregnancy and nursing periods. The daily additional liquid administered for 2 h to pregnant mothers consuming ethanol reached 16.6 ± 0.3 ml/day, Therefore, mothers consuming ethanol drunk 48.94 ± 2.77 ml of total liquid per day, similar to control mothers. Food consumption of the pregnant mothers and litter size was similar in both groups, with no significant difference in average maternal weight. In these conditions, mothers drank 8.1 ± 0.4 gr/kg/day of ethanol on average during the treatment period (Table 2). Since this value could be overestimated, at 7–15 days of pregnancy, maternal blood alcohol concentration (BAC) was measured 12 h after lights, were turned off reaching a concentration of 62 ± 22 mg/dl. Pup weight was not significantly altered between control and ethanol exposed rats at P1 or P7 (Table 2).

**Table 2:** Effects of forced ethanol (10%) consumption on rat dams and their offspring.

**Variable**	**Maternal treatment (&)**		
	**Ethanol**	**Water**	***P***
Maternal ethanol consumption (g ethanol/Kg/day)	8.5 ± 0.7	NA	
Maternal food consumption (g/kg/day)	87 ± 6.1	102 ± 4.8	
Maternal liquid consumption (ml/day)	34.8 ± 2.6	55.4 ± 2.8	^***^
Maternal additional water consumption (ml/day)	16.6 ± 0.3	NA	
Average of maternal weight (g)	306 ± 8.4	315 ± 8.1	
Offspring weight -postnatal 1 day (g)	6.8 ± 0.3	7.1 ± 0.2	
Offspring weight -postnatal 7–8 day (g)	14.5 ± 0.5	16.1 ± 0.7	
Male littermates (%)	50 ± 6.0	43 ± 5.3	
Female littermates (%)	50 ± 6.0	55.9 ± 5.3	
Litter size	13.6 ± 0.4	13.7 ± 0.5	

^&^*Both treatment include sucralose 64 mg/l. P, statistical probability*,

*** *p < 0.001. Analysis by Student's t-test. Mean ± SEM, N = 19–20. NA, not applicable*.

**Figure 1:**
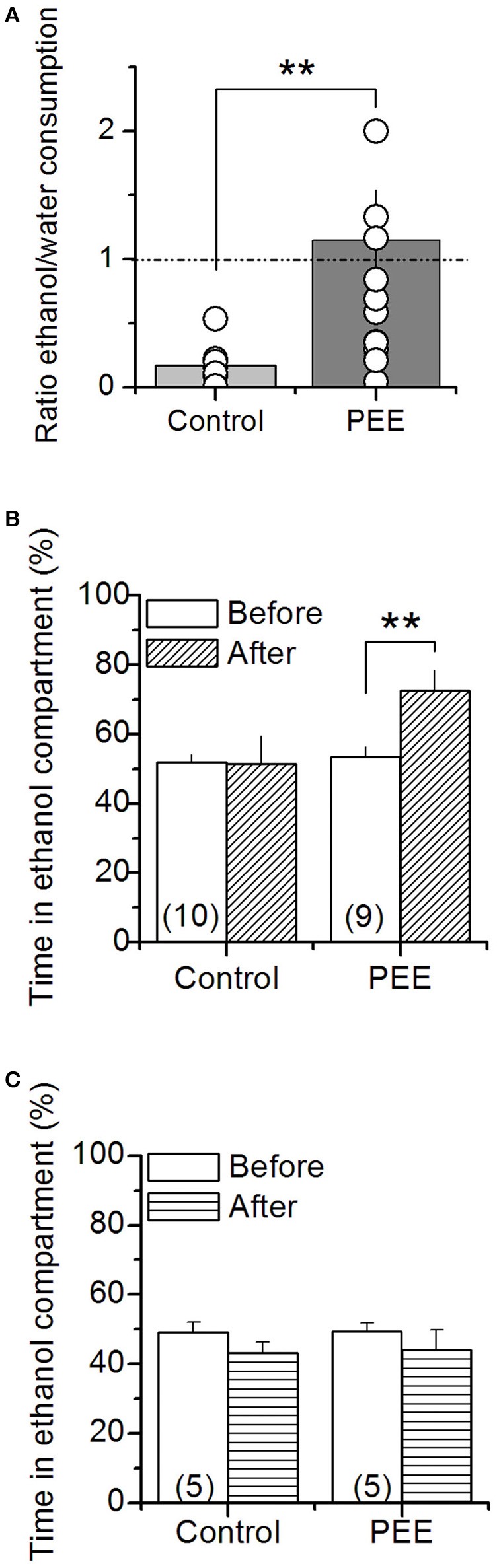
Ethanol consumption and ethanol-dependent conditioning in control and PEE rats. **(A)** Free choice of ethanol (10%) solution or water consumption was measured for 24 h in male rats. The graph shows the ratio between ethanol and water consumption, each point corresponds to one litter. Ethanol conditioning place preference was measured in male **(B)** and female **(C)** rats. *N* is indicated into the bars. ^**^P < 0.01 (unpaired Student's *t*-test).

To test whether the PEE model effectively induces addictive vulnerability, 70 day old offspring rats, we measured free choice ethanol consumption in male and conditioned place preference (CPP) in male or female offspring. We found that PEE rats had higher ethanol consumption compared to controls (Figure 1A), consuming 1.7 ± 0.5, compared with control 0.6 ± 0.2 gr/kg/day. Adult male rats also preferred the ethanol-conditioned context (Figure 1B). However, female rats of the same age did not present alcohol induced place preference (Figure 1C).

### PEE deregulates CAT, SOD1, and Gpx1 mRNA expression

**Figure 2:**
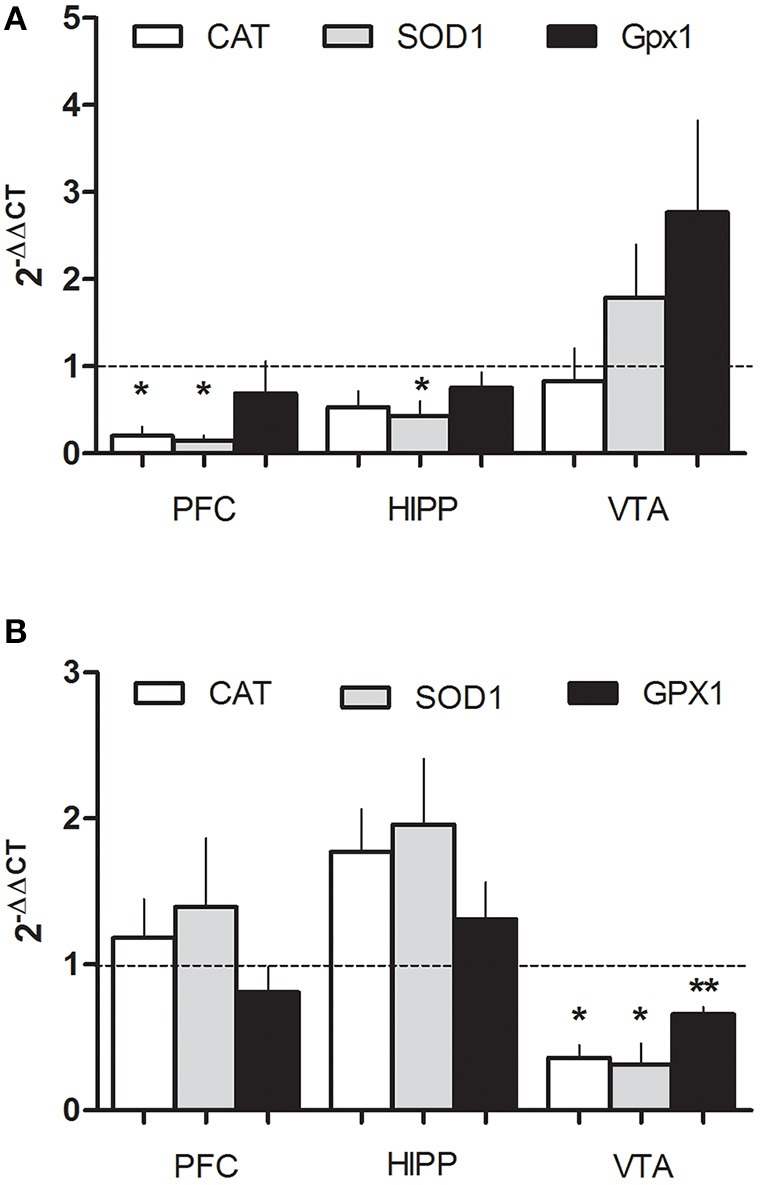
PEE modifies the expression of antioxidant enzyme mRNAs in rat brain. Total RNA was obtained as detailed in the methods section from the Prefrontal cortex, (PFC), Hippocampus (HIPP), and Ventral tegmental area (VTA) from control or PEE rats of 21 (P21) **(A)** or 70 (P70) **(B)** days old and amplified using RT-qPCR. Data are expressed as a fold change between PEE vs. control gene expression. *N* = 3–5 litters. ^*^P < 0.05, ^**^P < 0.01 (paired Student's *t*-test compared with 1).

To evaluate molecular changes in the general redox system induced by ethanol, specifically in regions involved with drug abuse disorders, we analyzed mRNA expression CAT, SOD1, and Gpx1 by RT-qPCR in 2 ages, P21 and P70. We observed significant differences depending on the region and age. At 21 days in the PFC, a drastic reduction in CAT mRNA (0.2 ± 0.1-fold change), and SOD1 (0.14 ± 0.06-fold change) were observed and SOD also decreased in HIPP (0.43 ± 0.17 fold of change; Figure 2A). In contrast, in older animals (P70) CAT and SOD 1-do not change in PFC or HIPP, however in the VTA, ethanol induced a robust decrease of CAT, SOD and Gpx1 mRNA (0.36 ± 0.09, 0.31 ± 0.15, 0.66 ± 0.05-fold change, respectively; Figure 2B). These results suggest that PEE induces differential deregulation of the redox homeostasis in the PFC, VTA, or HIPP in an age dependent manner, suggesting a low antioxidant power in the mesocorticolimbic pathway.

### PEE deregulates NOX2 expression

**Figure 3:**
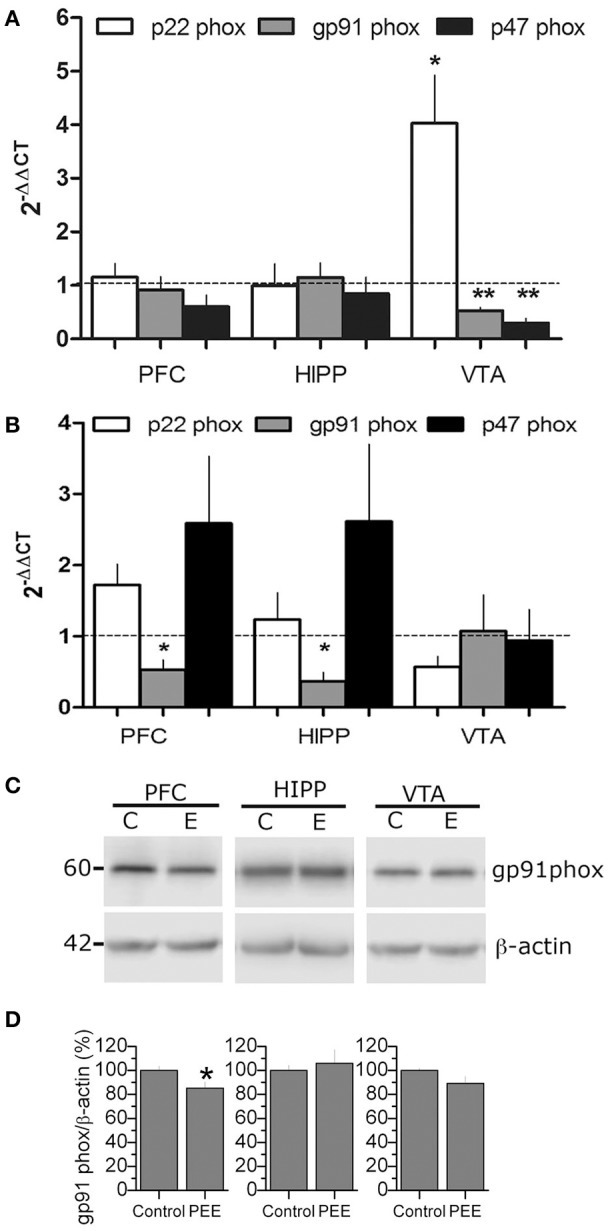
PEE modifies NOX2 mRNA expression in rat brains. **(A,B)** Total RNA was obtained from the Prefrontal cortex, (PFC), Hippocampus (HIPP), and Ventral tegmental area (VTA) from Control or PEE rats of 21 (P21) **(A)** or 70 (P70) **(B)** days old and amplified using RT-qPCR. Data are expressed as a fold change between PEE vs. control gene expression. *N* = 3–4 litters. ^*^P < 0.05, ^**^P < 0.01, paired Student's *t*-test compared with 1. **(C,D)** Total protein samples were obtained from control and PEE rats at P70. Immunodetection was performed using a gp91 phox specific antibody and normalized to β-actin. *N* = 9 litters. ^*^P < 0.05, ^**^P < 0.01 (unpaired Student's *t*-test).

Since NOX2 is an important source of superoxide implicated in learning and memory processes, we evaluated the expression of different components of NOX2 (i.e., p22 phox, p47 phox, and gp91 phox), by RT-qPCR; we also measured gp91 phox levels using western blot in P21 or P70 rats (Figure 3). Samples obtained from P21 rats showed a robust change in the VTA region where gp91 phox and p47 phox mRNA expression was significantly decreased (0.53 ± 0.06, 0.3 ± 0.09-fold changes, respectively; Figure 3A). In contrast, p22 phox mRNA expression increased compared to controls (4.0 ± 0.9-fold changes). At P70, gp91 phox mRNA levels significantly decreased in PEE rats in both PFC and HIPP (0.5 ± 0.14 and 0.4 ± 0.13-fold change compared to controls, respectively; Figure 3B). The gp91 phox protein levels are also reduced in PEE animals compared to controls, specifically in the PFC which also had low mRNA levels in this region (Figure 3C), suggesting that gp91phox expression could be controlled at the transcriptional level. Thus, these results show that PEE impacts NOX2 expression and could play a role in brain regions implicated in the reward signaling where antioxidant power appears to be low (Figure 2).

### PEE deregulates NMDAR expression

**Figure 4:**
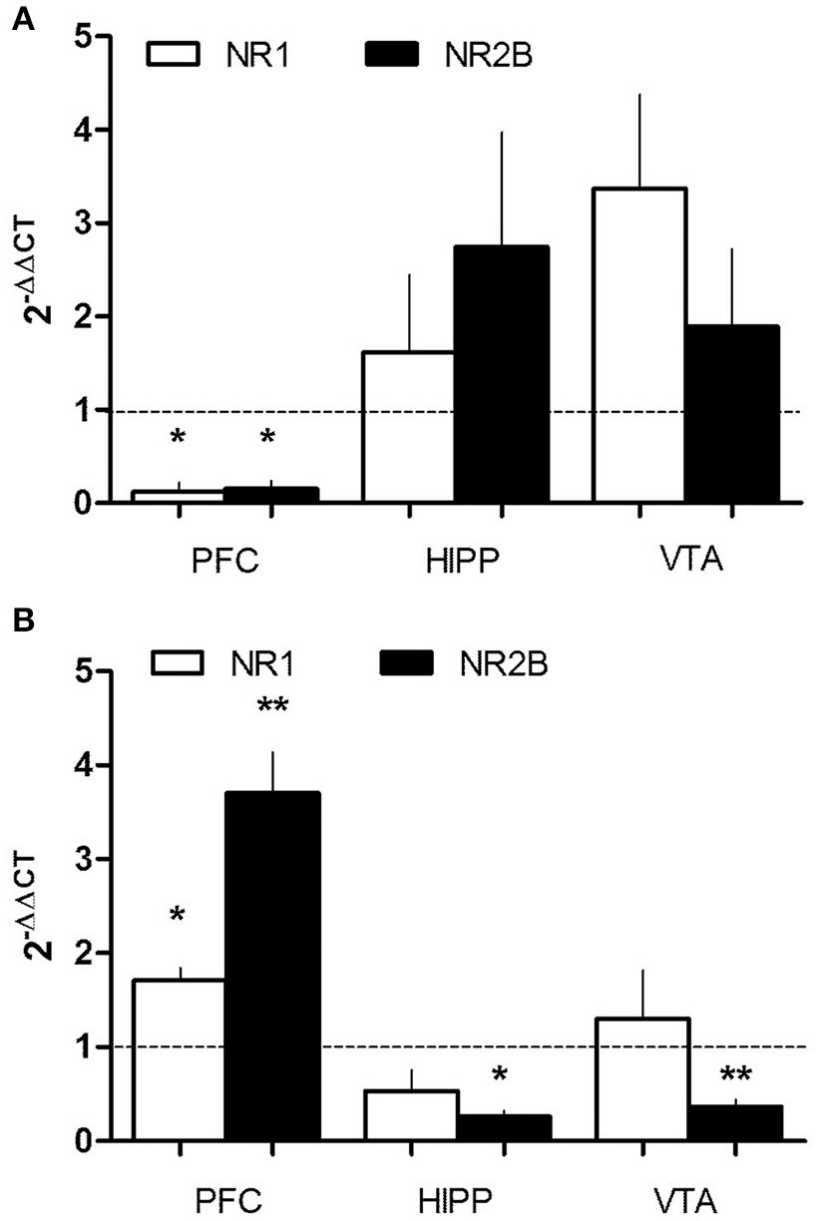
PEE modifies NMDAR mRNA expression in rat brains. Total RNA was obtained from the Prefrontal cortex, (PFC), Hippocampus (HIPP), and Ventral tegmental area (VTA) from Control or PEE rats of 21 (P21) **(A)** or 70 (P70) **(B)** days old. RT-qPCR was performed using specific primers for NMDAR mRNA subunits (NR1 and NR2B). Data is expressed as a fold of expression in PEE vs. control samples. *N* = 3–5 litters. ^*^P < 0.05, ^**^P < 0.01 (paired Student's *t*-test compared with 1).

Given that the reduction of gp91phox (mRNA and protein level) was more pronounced in the PFC, a region with glutamatergic activity, we evaluated if the glutamate-receptor NMDAR could be affected by PEE. mRNA levels of two NMDAR subunits, NR1 and NR2B, were analyzed by RT-qPCR in mesocorticolimbic brain areas. Interestingly, we found differential deregulation of NMDAR subunits in P21 and P70 PEE rats (Figures 4A,B). mRNA of both subunits NR1 and NR2B was significantly reduced in P21 PEE rats (0.13 ± 0.1 and 0.15 ± 0.1, respectively) in PFC, but no changes were observed in VTA or HIPP (Figure 4A). However, in P70 rats the NMDAR subunit levels were significantly increased (1.7 ± 0.13 NR1, 3.7 ± 0.4 NR2B) in the PFC, whereas NR2B mRNA levels were significantly decreased in the VTA and HIPP (0.4 ± 0.1 and 0.3 ± 0.1, respectively; Figure 4B). Thus, these results show that PEE induces changes in NMDAR mRNAs in both ages. It is particularly intriguing that in the PFC of P21 rats NMDAR mRNA expression is decreased, meanwhile in P70 rats it is increased. In association with the altered NOX2 expression (Figure 2), we speculate that prenatal ethanol consumption regulates NMDAR function by modifying the crosstalk between NMDAR-NOX2 complex at PFC.

### Inhibition of NOX 2 rescues alcohol-seeking behavior generated by PEE

**Figure 5:**
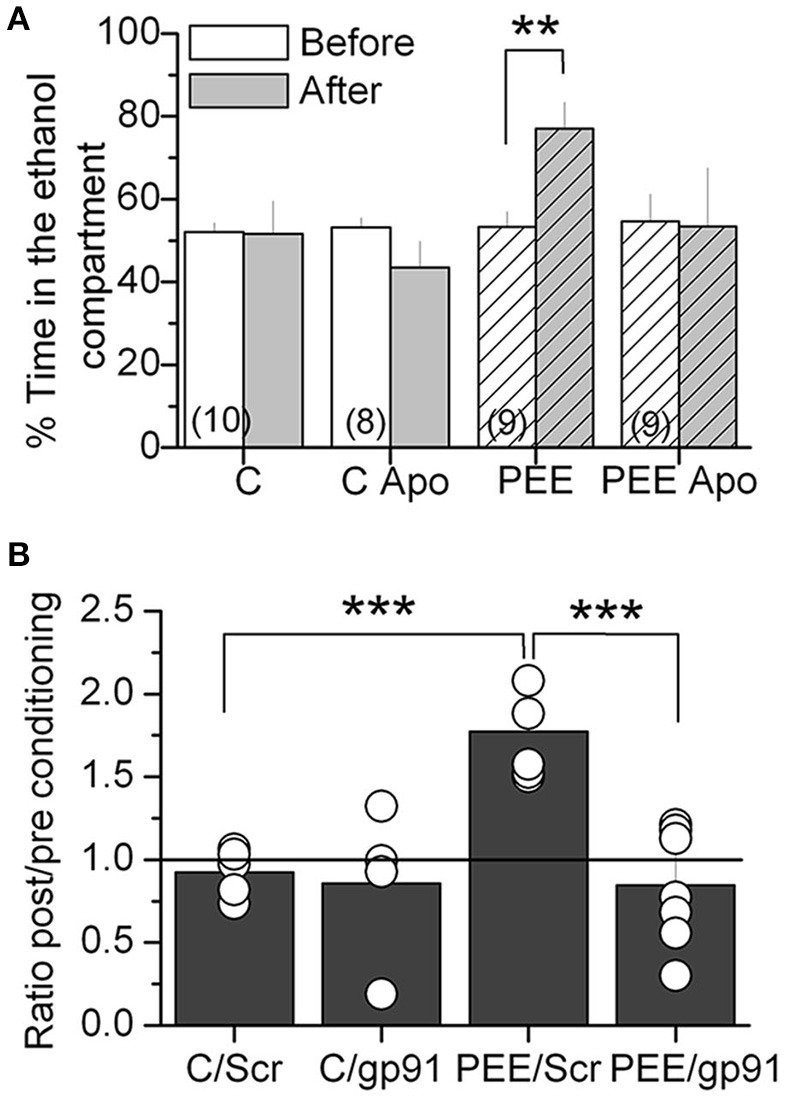
NOX 2 inhibition blocked ethanol-induced CPP in PEE rats. **(A)** Oral treatment with 5 mM of apocynin (Apo) reduced the ethanol-induced CPP. **(B)** Stereotaxic injection of gp91 ds-tat (gp91) and not scrambled (Scr) peptides into the VTA blocked ethanol-induced CPP. Data are expressed as a ratio of the time spent in the compartment where ethanol is injected after alcohol-paired training vs. preconditioning periods. ^**^P < 0.01 ^***^P < 0.01, Student's *t*-test.

Since alcohol seeking behavior is altered in P70 rats, we tested NOX2 function during ethanol preference behavior using a NOX2 inhibitor apocynin (Apo, 5 mM), which was administrated in drinking water for 1 month (P30-P70). Apo treatment blocked ethanol-conditioning behavior in P70 rats, reaching similar conditions to control animals, without affecting the behavior of control animals (Figure 5A). Next we used a molecular NOX2 inhibitor, a peptide gp91 ds–tat (gp91), which was specifically infused into the VTA through a cannula 20 min before exposure to the context during the CPP-test each day. Interestingly, we found that the peptide gp91 ds–tat and not the scrambled (Scr) peptide, abolished ethanol place preference (Figure 5B), suggesting that NOX2 has a local function into the VTA. All together these results strongly suggest that NOX2 controls neuronal activity in the VTA to increase ethanol-seeking behavior, probably impacting reward signaling in the mesocorticolimbic pathway, which might contribute to the reinforcing effects of alcohol.

## Discussion

Our results suggest that altered redox homeostasis along with NOX2 dependent neuronal activity are associated with augmented alcohol seeking behavior in adult PEE male animals.

We observed that PEE induced modifications to the gene expression of redox homeostasis proteins -CAT, SOD, Gpx1, and NOX2- and glutamatergic NMDARs, in an age and brain region dependent manner. NOX2 inhibition, specifically in the VTA, rescued seeking behavior in adult rats with the PEE condition. These findings suggest that PEE induces a neuronal activity-dependent hyperfunction of the VTA and hypofunction in the PFC mediated by NOX2, presumably influenced by neuronal redox state and NMDAR activity in each area, which are programed by PEE.

We described the effect of PEE as gender specific, since only male offspring were conditioned to ethanol in the CPP experiment. PEE Sprague-Dawley female rats are less sensitive to the glutathione-dependent mechanism than males, in regards to the expression of hippocampal long term plasticity (Patten et al., 2013). Additionally, gender specificity depends on the rodent species since PEE increased female, and not male, sensitivity to the rewarding effects of ethanol (Barbier et al., 2009; Torres et al., 2014). Future studies will evaluate the role of rodent species and redox state in female offspring, dependent drug conditioning.

Numerous studies have shown that drug abuse (chronic or acute) increases oxidative stress, and affect the function of S-glutathionylated proteins involved in neurotransmission and dendritic spine structure (for review see Uys et al., 2014; Womersley and Uys, 2016). Moreover, ROS are implicated in the development of behavioral sensitization following repeated cocaine exposure. Furthermore, increased ROS following cocaine exposure may be involved in reward signaling in the mesocorticolimbic pathway which is associated with reinforcing the effects of cocaine (Uys et al., 2014). To date, the role of ROS in vulnerable conditions was recently described, a lesion in the ventral hippocampus of neonatal rats (NVHL) can produce addiction vulnerability, as observed in long-term cocaine sensitization as well as self-administration of nicotine in schizophrenia rat models (Rao et al., 2016). Treatment of adolescent (P42) and adult NVHL rats with NAC, a potent anti-oxidant, reduced nicotine seeking behavior after nicotine self-administration (Rao et al., 2016). However, the specific source of ROS on addiction or vulnerability, as well as the molecular mechanism underlying the elevated ethanol seeking behavior in our model is unknown. Regarding with the context redox state, we found that several enzymes involved in ROS homeostasis, CAT, SOD1, Gpx11 are differentially down regulated in the VTA, PFC, or HIPP of PEE offspring at P21 or P70 (Figure 2) suggesting a persisting imbalance of redox homeostasis similar to that observed by Brocardo et al. (2011, 2017). Additionally we observed specific local regulation in the VTA, suggesting that ROS imbalance in Dopaminergic neurons (DA neurons) could be involved in seeking behavior. In this sense, PEE heightens dopaminergic activity in the VTA and alters the mesocorticolimbic pathway response to postnatal ethanol exposure (Fabio et al., 2015). Moreover, *in vitro* studies showed that ROS (specifically H_2_O_2_) modulate DA neuron activity (Avshalumov et al., 2007). Thus, an interesting mechanism that could partially explain seeking behavior in rats involves ROS imbalance, which is produced by down-regulation of anti-oxidant enzymes promoted by *in utero* ethanol exposure.

In spite of the role of ROS in addictive like behavior is widely supported (see reviews Uys et al., 2014; Womersley and Uys, 2016), the specific ROS source involved in vulnerable addictive brain has not been identified. The NOX2 complex is an interesting candidate because it modulates the glutamatergic pathway in a neuronal activity dependent manner, specifically through NMDAR modulation. NOX2/ROS and NMDAR are reciprocally and positively regulated in order to modulate synaptic plasticity and memory in the hippocampus (Kishida et al., 2005; Brennan et al., 2009; Massaad and Klann, 2011; Beckhauser et al., 2016). NOX2 participates in NMDAR activation (Kishida et al., 2005), and NMDAR activation stimulates NOX2; proposing NOX2 as a primary source of superoxide induced by NMDA in neurons (Brennan et al., 2009). Only one previous study showed that ethanol in acute high doses (2.9 gr/kg i.p.), at gestational day 9 (embryos analyzed 6 h after injection), regulates NOX2, and promotes teratogenesis, embryopathies, and neurodevelopmental deficit symptoms in mice embryos (Dong et al., 2010). We observed that PEE modified the levels of p22 phox, and p47phox, at P21 in VTA, while their expression did not change in PFC and HIPP in this age (Figure 3A). On the contrary to P21 (our results) and mice embryos (Dong et al., 2010), at P70 the gp91 phox subunit was down-regulated in HIPP and PFC regions (Figures 3B,C). Therefore, we propose that PEE promotes a persistent and differential regulation of NOX2 gene expression in the mesocorticolimbic pathway brain regions.

How can NOX2 modify the activity of mesocorticolimbic pathway brain regions to explain increased addictive like behavior? We propose an orchestrated function between NOX2 and NMDAR in the altered cellular redox state generated by PEE. This proposal is supported by previous studies concerning a functional communication between NOX2 and NMDAR in neuronal plasticity (Kishida et al., 2005; Brennan et al., 2009; Massaad and Klann, 2011; Beckhauser et al., 2016). Moreover, in addition to NOX, neuronal redox state also regulates the NMDAR opening (Aizenman et al., 1989; Sucher and Lipton, 1991), and in turn recent evidence showed that NMDAR activity is coupled with the up-regulation and enhanced activity of the glutathione system (Gpx1, 2, and 4) both in glia and neurons *in vitro* (Baxter et al., 2015). This coupling between NMDAR and glutathione could be affected by ethanol exposure *in utero*. Indeed, we found that PEE reduced the mRNA levels of both NMDAR subunits and antioxidant enzymes (described above) mostly in PFC at p21. In contrast, at P70 we found that both NMDAR mRNA subunits were upregulated while NOX2 mRNA and protein levels were downregulated (Compare Figures 3, 4). This inverse correlation was found only in the PFC at P70, but not in the VTA and HIPP.

The functional regulation of PFC is crucial for elaborate addiction like behavior, since PFC plays a key role in memory extinction induced by drug reinforcers (Millan et al., 2014). Also PEE reduced neural activity specifically in the infralimbic cortex of adolescents offspring (Fabio et al., 2015). Thus, we suggest that the redox state, NOX2, and NMDAR are part of the same mechanism, which impacts neuronal transmission and/or plasticity in the PFC as well as the VTA. Future studies will focus on understanding the effect of NOX2 on NMDAR function in the PFC of PEE animals.

The mechanism of how NOX2 integrates PFC hypofunction or VTA hyperfunction is unknown. There are several hypothetical mechanisms about how ethanol could participate in reward behavior through ROS and NOX2. Similar to other models, it will be interesting to test the possibility that during ethanol consumption, oxidative stress or ethanol itself could affect NOX2 activity and/or expression in interneurons -GABAergic parvoalbumin (PV) subtype- in the fetal brain, affecting both interneuron migration and GABAergic synaptic transmission in the PFC impacting, in turn, its intrinsic glutamatergic activity in this region (Skorput et al., 2015; Qiu et al., 2016). However, since our results show that gp91 phox, the most abundant NOX2 protein, does not change during early development (P21), it is likely that NOX2 has no relationship with the number of interneurons in the PFC. Another possibility is that high ROS levels, induced by downregulation of antioxidant enzymes in the VTA and unchanged NOX2 mRNA expression (Figures 2, 3), could modify NMDAR dependedent transmission to control DAminergic neuronal activity in the VTA area. In fact, NMDAR synaptic plasticity is increased by previous ethanol experiences (Bernier et al., 2011). Furthermore, DA neuron activation increases ROS production by inhibiting catalase, enhancing endogenous intracellular H_2_O_2_; allowing DA neuron excitability to be modulated in midbrain (Avshalumov et al., 2005). Nevertheless, ROS levels must be closely regulated, otherwise apoptosis in DA neurons and neurodegeneration can be induced (Choi et al., 2012; Hernandes et al., 2013).

Our PEE animal model presented increased ethanol seeking behavior compared to control rats, but lacked an evident FASD phenotype. Our results support the role of redox homeostasis in the pathophysiology of drug addiction and show, for first time, that NOX2 could be involved in alcohol seeking behavior in mammals, specifically in the vulnerability associated with problematic alcohol consumption. Further, our findings open a new avenue to investigate the specific NOX2-dependent neuronal mechanism along with its potential as a therapeutic target for treating alcohol and other drug addictions.

## Author contributions

MC and PH contributed to the design of the experiments, performed the experiments, and wrote the manuscript. PH and Ed supervised the project, designed the experiments and wrote the manuscript. SV, DM, and CG performed the experiments and revised the manuscript. All authors read and approved the final version of the manuscript.

### Conflict of interest statement

The authors declare that the research was conducted in the absence of any commercial or financial relationships that could be construed as a potential conflict of interest.

## Supplementary material

Supplementary Figure 1 Daily consumption of liquid during mother's treatment. Mothers were treated with water + sucralose (black dots) or ethanol 10%+sucralose (white dots) as is described in the methods section. Mothers that received ethanol treatment were also exposed to additional water consumption for 2 h every morning (gray dots). Dot line depicts the day of birth (E21). Mean ± SEM, *N* = 20.

## Abbreviations

HIPP: Hippocampus
VTA: Ventral Tegmental Area
PFC: Prefrontal Cortex
CAT: Catalase
SOD1: Cu/ZnSOD Superoxide dismutase
Gpx1: Glutathione peroxidase isoform 1
P21: Post natal day 21
P70: Post natal day 70.
