# Supplementary figures and images for: NADPH Oxidase Isoform 2 (NOX2) Is Involved in Drug Addiction Vulnerability in Progeny Developmentally Exposed to Ethanol
Source: Front Neurosci. 2017 Jun 14;11:338. doi: 10.3389/fnins.2017.00338 (PMC5469911; doi:10.3389/fnins.2017.00338)

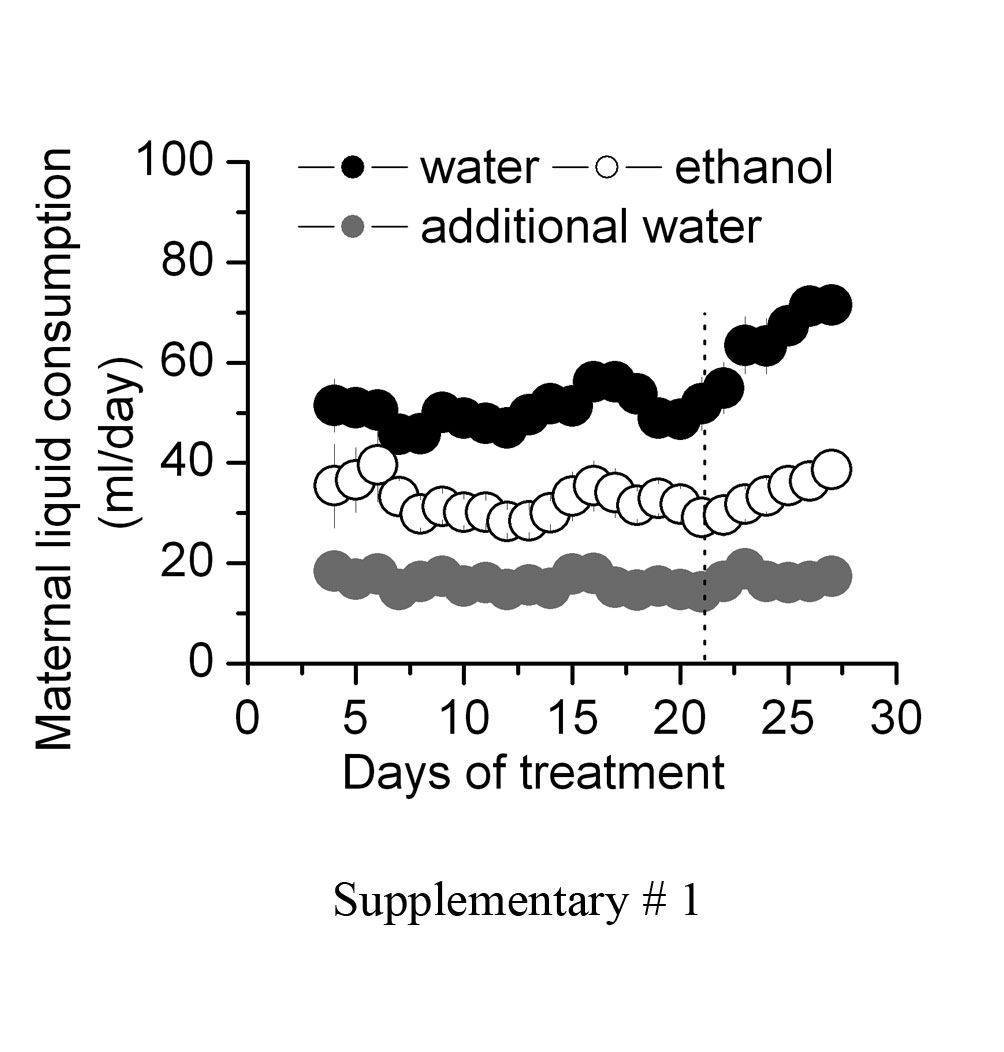

Supplement: Supplementary Figure 1 — Daily consumption of liquid during mother's treatment. Mothers were treated with water + sucralose (black dots) or ethanol 10%+sucralose (white dots) as is described in the methods section. Mothers that received ethanol treatment were also exposed to additional water consumption for 2 h every morning (gray dots). Dot line depicts the day of birth (E21). Mean ± SEM, N = 20. [file Image1.JPEG]
